# The Disease-Specific Phenotype in Cardiomyocytes Derived from Induced Pluripotent Stem Cells of Two Long QT Syndrome Type 3 Patients

**DOI:** 10.1371/journal.pone.0083005

**Published:** 2013-12-11

**Authors:** Azra Fatima, Shao Kaifeng, Sven Dittmann, Guoxing Xu, Manoj K. Gupta, Matthias Linke, Ulrich Zechner, Filomain Nguemo, Hendrik Milting, Martin Farr, Jürgen Hescheler, Tomo Šarić

**Affiliations:** 1 Institute for Neurophysiology, Medical Center, University of Cologne, Cologne, Germany; 2 Heart and Diabetes Center NRW, University Hospital of the Ruhr-University of Bochum, Bad Oeynhausen, Germany; 3 Erich & Hanna Klessmann-Institute for Cardiovascular Research and Development, Bad Oeynhausen, Germany; 4 Institute for Human Genetics, Johannes Gutenberg University, Mainz, Germany; University of Tampere, Finland

## Abstract

Long QT syndromes (LQTS) are heritable diseases characterized by prolongation of the QT interval on an electrocardiogram, which often leads to syncope and sudden cardiac death. Here we report the generation of induced pluripotent stems (iPS) cells from two patients with LQTS type 3 carrying a different point mutation in a sodium channel Na_v_1.5 (p.V240M and p.R535Q) and functional characterization of cardiomyocytes (CM) derived from them. The iPS cells exhibited all characteristic properties of pluripotent stem cells, maintained the disease-specific mutation and readily differentiated to CM. The duration of action potentials at 50% and 90% repolarization was longer in LQTS-3 CM as compared to control CM but this difference did not reach statistical significance due to high variations among cells. Sodium current recordings demonstrated longer time to peak and longer time to 90% of inactivation of the Na^+^ channel in the LQTS-3 CM. This hints at a defective Na^+^ channel caused by deficiency in open-state inactivation of the Na^+^ channel that is characteristic of LQTS-3. These analyses suggest that the effect of channel mutation in the diseased CM is demonstrated *in vitro* and that the iPS cell-derived CM can serve as a model system for studying the pathophysiology of LQTS-3, toxicity testing and design of novel therapeutics. However, further improvements in the model are still required to reduce cell-to-cell and cell line-to-cell line variability.

## Introduction

The long QT syndrome (LQTS) is a rare inborn heart condition in which delayed repolarization of the heart following a heartbeat increases the risk of episodes of *torsade de pointes* [1]. These episodes may lead to palpitations, fainting and sudden death due to ventricular fibrillation. Episodes may be provoked by various stimuli, such as exercise, emotion, loud noise, swimming, depending on the subtype of the condition. LQTS can be genetic or acquired in nature. The prevalence of the disease is around 1/5,000 in the general population [2,3]. 20% of patients with syncope who remain untreated die within one year and 50% die within 10 years [4]. The type 3 of the long QT syndromes (LQTS-3) is caused exclusively by gain-of-function mutation in the *SCN5A* gene that encodes for the alpha subunit of the Na^+^ channel Na_v_1.5. A large number of mutations in all domains of Na_v_1.5 have been characterized as leading to or predisposing to LQTS-3 [3]. Most of these mutations produce increased persistent sodium current (I_Na_) during the plateau and repolarization phase of the action potential (AP) due to defective open-state inactivation of the Na_v_1.5 channel. This defect, in addition to other gain-of-function gating defects in mutant Na_v_1.5 channels, leads to prolonged repolarization of the ventricle and prolonged QT interval on an ECG [5,6]. Mutations in *SCN5A* can also lead to a loss-of-function of the Na_v_1.5 channel and they are associated with several other genetically heterogeneous disorders including Brugada syndrome (BrS), cardiac conduction disease, sick sinus syndrome sudden infant death syndrome and others [7-9].

The functional characteristics of *SCN5A* mutations have been analyzed by ectopic expression of mutant Na_v_1.5 channels in mammalian cell lines as well as in transgenic mouse model systems [10,11]. However, due to the distinct electrophysiological properties of cardiomyocytes (CM) derived from mice and humans, human CM are required for investigating the pathogenesis of disease, designing patient specific therapies and identifying cardiotoxic substances. 

Induced pluripotent stem (iPS) cells carry the developmental potential of human embryonic stem (ES) cells and have been heralded as a powerful tool to study development and disease. These cells can serve as a source of human diseased CM *in vitro* providing a platform to study the disease pathology and design drug treatments if CM are able to recapitulate the disease phenotype. The proof of principle for this paradigm has been provided by recent publications in iPS cell-based murine models of LQTS-3 and an overlap syndrome of Na_v_1.5 related cardiac disease [12,13]. In the human model, it has been shown that iPS cells derived from a patient with LQTS-1 [14] or LQTS-2 [15-17] can differentiate to CM which respond appropriately to clinically relevant drugs and recapitulate the disease phenotype and the channel specific physiological dysfunction. Here, we describe two *SCN5A* mutations (p.V240M and p.R535Q) in two LQTS-3 patients and show that CM obtained from LQTS-3 patient-derived iPS cells exhibit the electrophysiological abnormalities associated with this disease. 

## Materials and Methods

### Reprogramming and cell culture

Skin biopsies from patients were collected with informed consent and approved protocol. We confirm that informed consent from all donors of skin biopsies was obtained for use of samples in research. The participants provided their written informed consent to participate in this study. The study was approved by the Ethics Committee of the Ruhr-University Bochum in Bad Oeynhausen, Georgstr. 11, 32545 Bad Oeynhausen, Germany, Registration No.: 41/2008. After surgical removal of epidermis, dermis was placed on culture dishes and fibroblasts migrating out of the explants were expanded. At third passage, 2x10^4^ cells were infected with a combination of pMXs-based retroviruses (Addgene, plasmid IDs: 13366, 13367, 13370, 13375) encoding the human transcription factors OCT3/4, SOX2, KLF4, and c-MYC [18]. The fibroblasts were transduced in the presence of 4 μg/ml polybrene (Sigma-Aldrich, Taufkirchen, Germany) for 8 hrs per day in two consecutive days and maintained in high glucose Dulbeco’s modified essential medium (DMEM) supplemented with Glutamax, 10% fetal bovine serum (FBS), 1% non essential amino acids (NAA), 1x Pen/Strep (100 U/ml penicillin, 100 μg/ml streptomycin), and 0.1 mmol/L ß-mercaptoethanol (βME). All cell culture reagents were purchased from Invitrogen (Life Technologies, Darmstadt, Germany) if not stated otherwise. Six days after the first transduction, 10,000 cells were plated on 0.1% gelatin-coated 60 mm dishes containing 5x10^5^ irradiated CF1 murine embryonic fibroblasts (MEF). From day 7, cells were maintained in DFBS medium (DMEM/F12 supplemented with Glutamax, 20% FBS, 1% NAA, 1x Pen/Strep, 0.1 mmol/L βME and 50 ng/ml basic fibroblasts growth factor (bFGF, Peprotech, Hamburg, Germany), 20 μg/ml vitamin C (Sigma-Aldrich) and 1 mM valproic acid (Sigma-Aldrich). ES cell-like colonies appeared between day 21-30 after the first infection. Putative iPS cell colonies were picked and expanded for subsequent validation. Established iPS cell lines were maintained on MEF in DMEM/F12 medium supplemented with Glutamax, 20% knockout serum replacer, 1% NAA, 0.1 mmol/L βME and 100 ng/ml bFGF. Cells were passaged by manual dissection of cell clusters every 5-6 days at the split ration of 1:3. Healthy iPS cell line that was described previously [19] and human ES cell line H1 (WiCell Research Institute, Madison, WI, USA) were used as control. Work with human ES cells has been approved by the regulatory authorities at the Robert Koch Institute, Berlin, Germany (permit number 1710-79-1-4-2-A10).

### Cardiac differentiation

Cardiac differentiation of human iPS and ES cells was carried out on the murine visceral endoderm-like cell line END2 [20]. Briefly, END2 cells [21] which were kindly provided by Christine Mummery (Leiden University Medical Center, The Netherlands) were mitotically inactivated for 3 hours with 10 μg/ml mitomycin C (Sigma-Aldrich) and 1.2×10^6^ cells were plated on 60 mm tissue culture dishes one day prior to starting the co-culture. To initiate the differentiation, undifferentiated ES and iPS cell colonies were detached from the feeder layers using cell scrapers (TPP, Trasadingen, Switzerland) and triturated into small clumps of cells using a pipette tip. The clumps were collected into a 50 ml Falcon tube and plated on the prepared END-2 monolayers in knockout-DMEM containing 1 mM L-glutamine, 1% NAA, 0.1 mmol/L βME and 1x Pen/Strep. The co-culture was left undisturbed at 37°C/5% CO_2_ for 5 days. First medium change was performed on day 5 and later on days 9, 12 and 15 of differentiation. CMs were used for experiments between days 20-30 of differentiation.

### RT-PCR and quantitative RT-PCR

Total RNA was isolated using TRIzol Reagent (Life Technologies) from undifferentiated iPS or ES cells as well as from beating areas derived from control ES and iPS cells and LQTS-3 iPS cells microdissected at day 25 of differentiation. DNase I-treated total RNA (500 ng) was reverse-transcribed using Superscript II RTase and random hexamers (Life Technologies). cDNA was diluted 1:4 with sterile tri-destilled water and 5 μl were amplified using JumpStart™RedTaq ReadyMix™ PCR Reaction Mix (Sigma). Negative controls were generated in RT reactions in which all reaction components were included except RTase. Reactions were terminated at the exponential phase of amplification and products were analyzed by agarose gel electrophoresis. For quantitative RT-PCR the cDNA probes were diluted 1:40 and 2 μl was amplified using SYBR Green PCR Master Mix (Qiagen, Hilden, Germany) in triplicate for each sample and each gene. Real-time PCRs were performed in a 7500 Fast System Real Time Cycler (Applied Biosystems, Carlsbad, CA, USA) and analyzed with SDS Shell 1.4 software (Applied Biosystems). *GAPDH* was used for normalization of expression levels of individual genes. 

### Immunocytochemistry

Undifferentiated ES and iPS cells were fixed with 4% paraformaldehyde (PFA), permeabilized with 0.1% Triton X-100 and blocked with 5% FBS and then stained overnight at 4°C with primary antibodies specific for OCT4 (Santa Cruz Biotechnology, Heidelberg, Germany, Cat. No. sc-5279, 1:100), SOX2 (Stemgent, Cambridge, MA, USA, Cat. No. 09-0020, 1:100), Nanog (R&D Systems, Wiesbaden, Germany, Cat. No. AF1997, 1:100), TRA-1-60 (BD Biosciences, Heidelberg, Germany, Cat. No. 560121, 1:100), TRA-1-80 (Santa Cruz, Cat. No. Sc-21706, 1:200) and SSEA4 (Santa Cruz, Cat. No. Sc-21704, 1:800). Samples were visualized after staining with AlexaFluor 488- or AlexaFluor 555-conjugated secondary antibodies (Life Technologies) for 1 hour at room temperature. Nuclei were counterstained with Hoechst 33342. Samples were embedded in ProLong Gold Antifade Reagent (Life Technologies) and observed on Axiovert Microscope (Carl-Zeiss Microimaging, Oberkochen, Germany) equipped with the image processing software Axiovision 4.5.

Beating areas from differentiations of ES and iPS cultures were microdissected on day 25 of differentiation and dissociated into single cells by trypsinization. The single CM were then plated onto μ-dishes^35 mm, high^ (Ibidi GmbH, Munich, Germany) coated with 2.5 μg/ml fibronectin. Three to five days after plating, the cells were fixed with 4% PFA and later stained as described above with anti-sarcomeric alpha-actinin (Sigma-Aldrich, clone EA-53, 1:400). Signals were visualized by AlexaFluor 555-conjugated secondary antibodies.

### Bisulphite pyrosequencing

Genomic DNA was isolated using the DNeasy Blood and Tissue Kit (Qiagen) and bisulfite treatment was performed using the EpiTect Bisulfite Kit (Qiagen). The methylation status of the promoters of *OCT4* and *NANOG* was analyzed on a PSQTM 96MA Pyrosequencing System (Biotage, Uppsala, Sweden) with the PyroGold SQA reagent kit (Biotage) [22]. Primers for bisulfite pyrosequencing were published previously [19]. Pyro Q-CpG software (Biotage) was used for data analysis.

### Recording of action potentials

Action potentials (AP) of spontaneously beating single CM were measured by means of the whole-cell current clamp technique using an EPC-9 amplifier and the PULSE software package (Heka Elektronik, Lambrecht, Germany). Single CM were obtained by dissociating microdissected beating areas at day 20-30 of differentiation with collagenase B. Cells were plated on fibronectin (2.5 μg/ml)-coated glass cover slips and incubated for 48 hours prior to measurements. Cell membrane capacitance was determined on-line using the Pulse program (Heka Elektronik). The experiments were performed at a bath temperature of 37±1°C in standard extracellular solution containing (in mM) 140 NaCl, 5.4 KCl, 1.8 CaCl_2_, 1 MgCl_2_, 10 HEPES and 10 glucose (pH was adjusted to 7.40 at 37°C with NaOH). Patch-clamp pipettes were filled with intracellular solution containing (in mM) 50 KCl, 80 K-aspartate, 1 MgCl_2_, 3 MgATP, 10 EGTA and 10 HEPES (pH was adjusted to 7.40 with KOH). All electrophysiological data was analyzed off-line with custom made analysis software. Cardiomyocytes were classified into ventricular-, atrial- and pacemaker/nodal-like cells according to visual inspection of AP morphology and the quantitative criteria that are summarized in the Table S1 in File S1.

### Voltage clamp recordings

Voltage-gated Na^+^ and L-type Ca^2+^ currents were recorded using extracellular solution containing (in mM): NaCl 120, KCl 5, CaCl_2_ 3.6, MgCl_2_ 1, tetraethyl ammonium (TEA) chloride 20, HEPES 10, pH adjusted to 7.40 at 37°C with TEA-OH. Intracellular solution contained (in mM): CsCl 120, MgCl_2_ 3, MgATP 5, EGTA 10, HEPES 5, pH adjusted to 7.40 with CsOH. All substances were, if not stated otherwise, obtained from Sigma-Aldrich Chemie GmbH. Na^+^ channel currents were elicited by a family of 100-ms depolarizations from a –90 mV holding potential (HP) to voltages ranging from –60 to +55 mV in 5-mV steps. To estimate the Ca^2+^ current contamination in Na^+^ currents the L-type Ca^2+^ currents were co-measured in the cells where Na^+^ channel currents were recorded. Ca^2+^ channel currents were measured with a double-pulse protocol. First, a 100-ms depolarization from a HP of –90 mV to –40 mV was applied to inactivate Na^+^ and T-type Ca^2+^ channels, then L-type Ca^2+^ channels were elicited by a family of 67-ms depolarizations to voltages ranging from –60 to +50 mV in 10-mV steps. In Na^+^ and Ca^2+^ channel experiments, leak subtraction -P/4 protocol was applied from HP of –90 mV. When the maxima of Na^+^ and Ca^2+^ current amplitudes obtained from the same cell at corresponding potentials were compared, the mean contamination of Na^+^ currents with an L-type current was less than 1% and in the most relevant voltage range from –50 to –10 mV it was less than 0.5%. Considering that the activation of L-type Ca^2+^ channels is 5-10 times slower than the Na^+^ channel activation, we estimated the L-type current contribution to less than 0.1%. Thus, the Ca^2+^ current contamination in the Na^+^ currents was neglectable. The time to 90% inactivation of Na^+^ current was determined manually. First, the potential eliciting maximal Na^+^ channel current was determined for every cell. Then, the time that elapsed from the point where current amplitude reached its maximal value to the point where the current reached 10% of its maximal value was determined. Since the classical persistent current in cardiomyocytes with LQTS3-associated SCN5A mutations is typically smaller than 5% of the transient current we have assumed that at this point of measurement no classical persistent current was affecting the value of the measured “time to 90% inactivation” parameter. In all experiments, leak subtraction was applied. PULSE software, Excel (Microsoft, USA), Sigma Plot (SPSS Inc., Chicago, USA), CorelDraw (Corel GmbH, Deutschland), ORIGIN (Microcal Software, Northampton, USA) programs were used for displaying and analyzing the recordings. 

### Statistics

Data are presented as the mean ± standard error of the mean (SEM). Student’s t-test was applied for statistical evaluation; the significance level was set at p<0.05.

## Results

### Patient characteristics

Skin biopsy was obtained from two patients diagnosed with the LQTS-3 syndrome (Table 1). The criteria for BrS or an overlap syndrome were not met in a diagnostic work-up of any of the two patients. Patient 1 (NP0012) was a 32 year old female who presented with several episodes of *torsades de pointes* tachycardia after acute appendicitis and appendectomy and was diagnosed with the LQTS-3. While the potassium channels IK_r_ (KCNH2/KCNE2) and IK_s_ (KCNQ1/KCNE1) were free of disease causing variants, heterozygous mutation c.1604GA was found leading to a point mutation p.R535Q in *SCN5A* gene in the intracellular linker region between domains I and II (DI/DII) of Na_v_1.5 channel (Figure S1 in File S1). The 12-lead ECG demonstrated a QT duration of 500 ms after correction for heart rate (QTc) according to the Bazett's formula (Figure S2A in File S1). Patient 2 (NP0016) was a 30 year old male patient who also presented with recurrent syncopes and was diagnosed with the LQTS-3 syndrome carrying heterozygous mutation c.718GA in the *SCN5A* gene that causes the amino acid change p.V240M in the cytoplasmic loop between membrane-spanning segments four and five within the first domain (DI-S4/S5) of Na_v_1.5 channel (Figure S1 in File S1). The 12-lead ECG recorded a QTc of 450 ms (Figure S2B in File S1). Since common *SCN5A* polymorphisms, such as variant allele c.1673AG present in 19-24% of healthy population that leads to a substitution of amino acid histidine (H) with arginine (R) (p.H558R) in Na_v_1.5 may affect function of wild-type channels [23] and also those with coexisting mutations [24,25], we have determined whether this polymorphism is present in genomic DNA of patients and control iPS cells. These analyses revealed that this polymorphism was present as a heterozygous allele in all samples (Figure S3 in File S1), suggesting that if any modulation of Na_v_1.5 function would occur through this polymorphism the effect would be manifested in CM derived from both control and LQTS-3 iPS cells and is thus not expected to be a confounding factor in our experiments.

**Table 1:** Characteristics of LQTS-3 patients reported in this study.

**Parameters**	**Patient NP0012**	**Patient NP0016**
Mutation and location	c.1604GA; p.R535Q in the intracellular linker region between DI and DII	c.718GA; p.V240M in the cytoplasmic loop between S4 and S5 within the DI
Gender	Female	Male
Date of birth	1972	1976
QTc (ms)	540	440
Clinical phenotype	At age of 32, the patient presented with several episodes of syncopes and *torsades de pointes* tachycardia, and survived one sudden cardiac death event. This patient developed DCM* in the later course of the disease.	This patient presented with frequent syncopes at age of 23 and at age of 30 with recurrent tachycardias. The patient was resuscitated from one sudden cardiac death event.
Therapy	Class IB anti-arrhythmic drug (mexiletine); β1-adrenergic antagonist (metoprolol); K^+^ and Mg2**^*+*^** supplementation; ICD implantation. Despite medication the ICD recorded frequent VES and terminated recurrent VT.	Antiarrhythmic drug (phenytoin); β1-adrenergic antagonist (metoprolol); K^+^ and Mg2**^*+*^** supplementation; ICD implantation. Despite medication episodes of *torsades de pointes* VT recurred and were terminated adequately by the ICD.

Abbreviations: QTc – corrected QT interval; DCM – dilated cardiomyopathy; ICD - implantable cardioverter defibrillator; VES - ventricular extrasystoles; VT – ventricular tachycardias.

^*^ Mutations in typical DCM-causing genes could not be identified in this patient.

### Characterization of iPS cells

**Figure 1:**
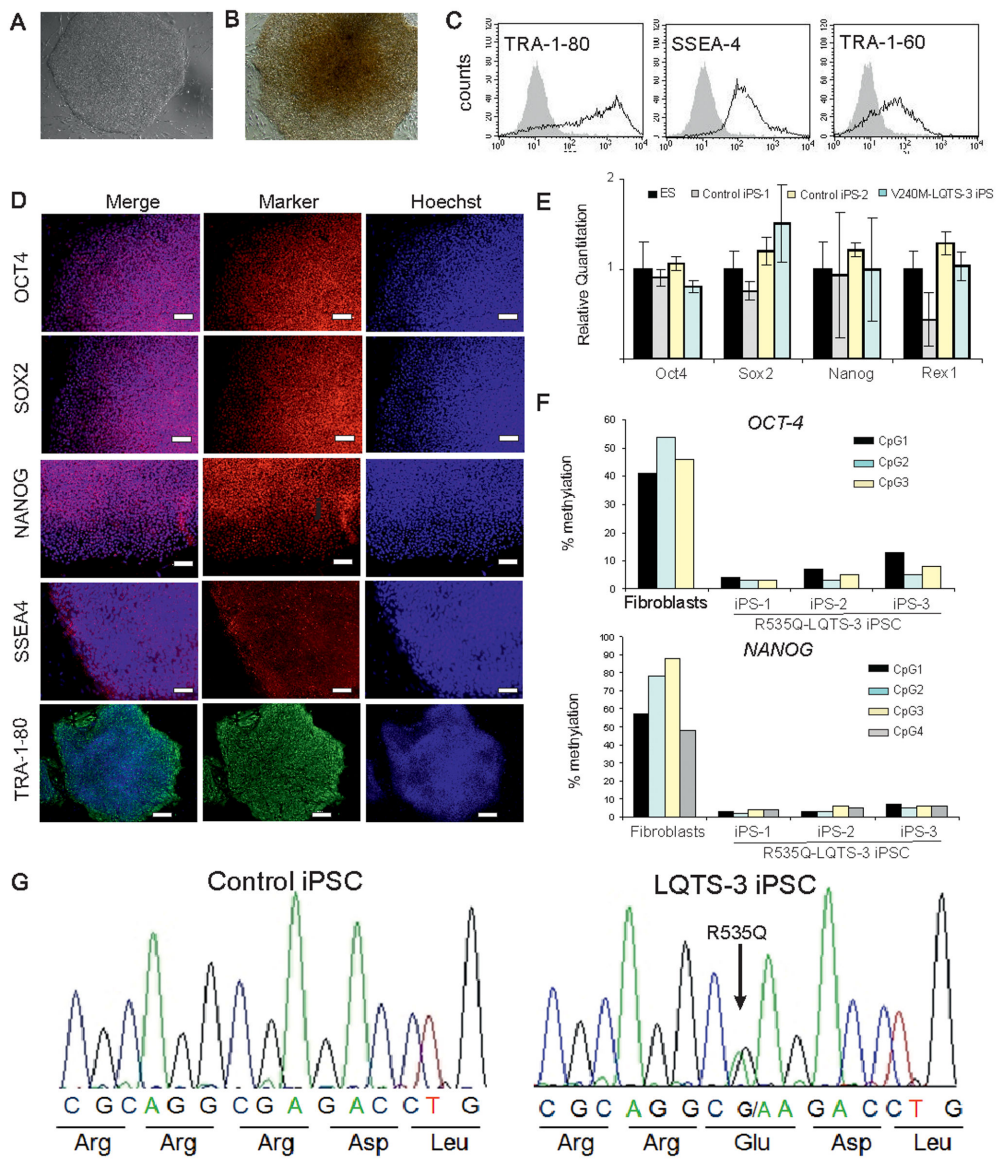
Characterization of LQTS-3 iPS cells. V240M-LQTS-3 iPS cell colony derived from patient NP0016 showing typical human ES cell-like morphology (A) and positive staining for alkaline phosphatase (B). The V240M-LQTS-3 iPS cell colonies expressed pluripotency markers OCT4, SOX2, NANOG, SSEA4, TRA-1-80 and TRA-1-60 as determined by flow cytometry (C) and immmunostaining (D). Expression of pluripotency markers in V240M-LQTS-3 iPS cells by qRT-PCR. Expression values were normalized to GAPDH and are presented as mean +SEM (n=3) relative to corresponding transcript levels in human ES cells (E). Methylation levels of promoter regions of OCT4 and NANOG genes in three different clones of R535Q-LQTS-3 iPS cells at passage 7 and R535Q-LQTS-3 fibroblasts (F). The LQTS-3 iPS colonies derived from dermal fibroblasts of patient NP0012 were verified for the presence of a heterozygous *SCN5A* point mutation p.R535Q by DNA sequencing (G).

Dermal fibroblasts were infected with retroviruses encoding for OCT4, SOX2, KLF4 and c-MYC to generate iPS cells. Three clones from patient NP0012 and one clone from patient NP0016 were obtained and used in this study. iPS cell colonies exhibited morphology similar to those of human ES cells (Figure 1A) and were stained positive for alkaline phosphatase (Figure 1B). LQTS-3 iPS cells expressed endogenous pluripotency markers at the protein level as demonstrated by flow cytometry (Figure 1C, Figure S4A,B in File S1) and immunocytochemistry (Figure 1D, Figure S4C,D in File S1). At the transcript level, the expression level of endogenous pluripotency genes was comparable to that of control ES and iPS cells as shown by quantitative RT-PCR (Figure 1E) and the retrovirally encoded genes were silenced (Figure S5 in File S1). The methylation pattern in the promoter regions of *OCT4* and *NANOG* genes revealed the unmethylated status of these promoters in iPS cells indicating activation state of the endogenous genes in contrast to fibroblasts which showed high degree of methylation in these regions (Figure 1F). Furthermore, the LQTS-3 iPS cell lines from both patients were found to carry the expected *SCN5A* gene mutation but not the control cell line (Figure 1G, Figure S6 in File S1).

### Cardiomyocyte differentiation

**Figure 2:**
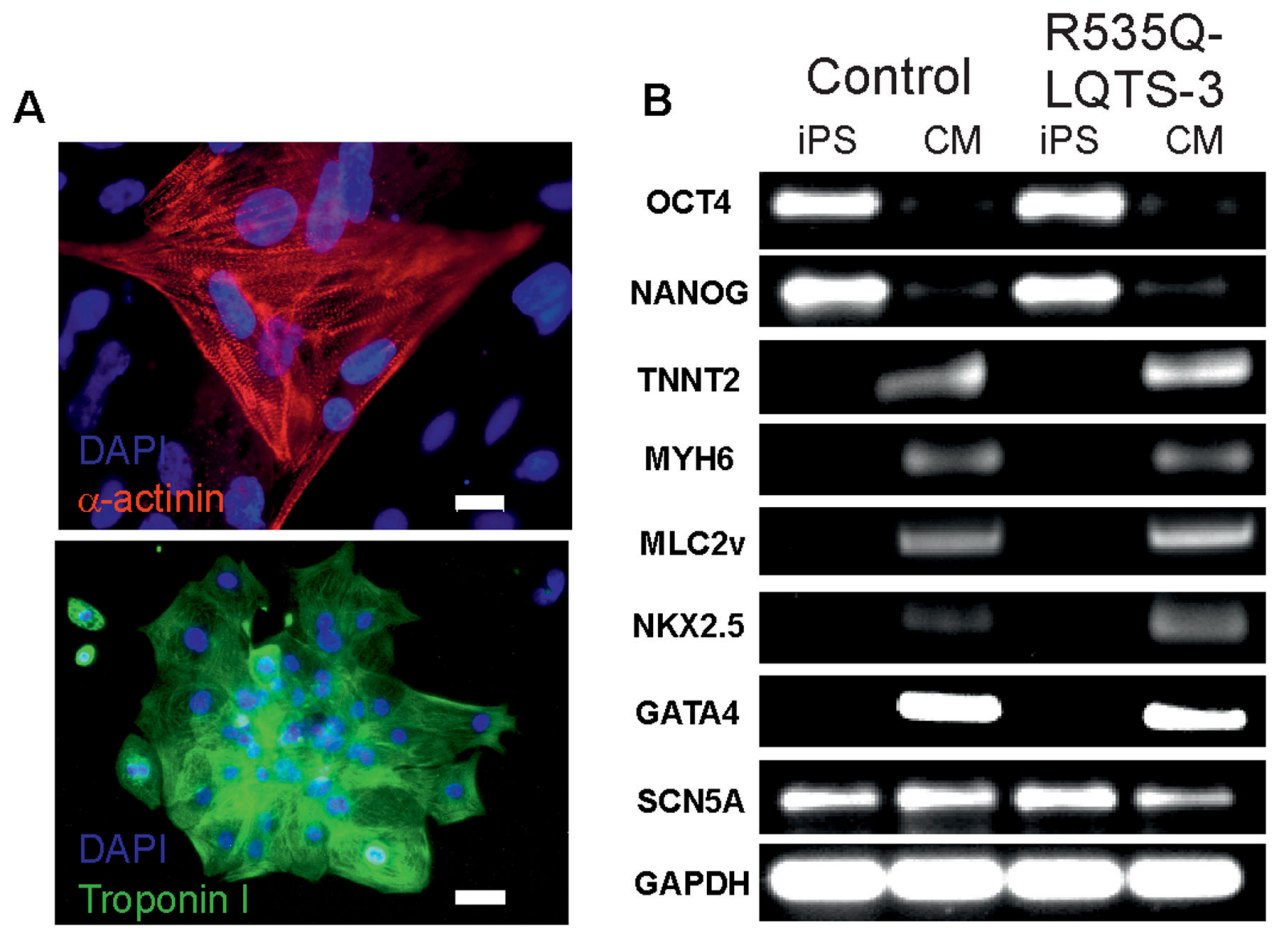
Expression of cardiac markers in R535Q-LQTS-3 iPS cell-derived CM. (A) Immunocytochemical analysis of cardiac markers α-actinin (red) and troponin I (green). Nuclei were counterstained with DAPI (blue). Scale bars: 20 μm (α-actinin) and 100 μm (troponin I). (B) The analysis of expression levels of cardiospecific transcripts was done by RT-PCR showing that the R535Q-LQTS-3 iPS cell-derived cardiomyocytes express cardiac transcripts at a similar level as their human ES cell-derived counterparts.

All iPS and ES cell lines showed comparable cardiac differentiation potential giving rise to spontaneously contracting areas after 8-9 days of differentiation on END-2 cells. The LQTS-3 CMs expressed cardiac proteins α-actinin and troponin I and exhibited typical cross-striations (Figure 2A). Microdissected beating clusters (BCs) derived from control cell lines and LQTS-3 iPS cells expressed comparable levels of transcripts encoding for cardiac proteins troponin T (*TNNT2*), NK2 homeobox 5 (*Nkx2.5*), myosin light chain ventricular (*MLC2v*), GATA binding protein 4 (*GATA4*), alpha myosin heavy chain (*MYH6*) and sodium channel Na_v_1.5 (*SCN5A*) (Figure 2B). These transcripts were not detected in undifferentiated iPS cells, with the exception of *SCN5A*, which were expressed at the same level in iPS cells and CM (Figure 2B). The pluripotency genes *OCT4* and *NANOG* were expressed at high levels in undifferentiated cells and were present only at very low levels in beating areas, most likely due to the presence of some residual pluripotent iPS cells (Figure 2B). These data suggest that beating areas isolated from differentiating LQTS-3 iPS cells contain CM with features similar to CM from control cell lines. 

### AP parameters in LQTS-3 cardiomyocytes

**Figure 3:**
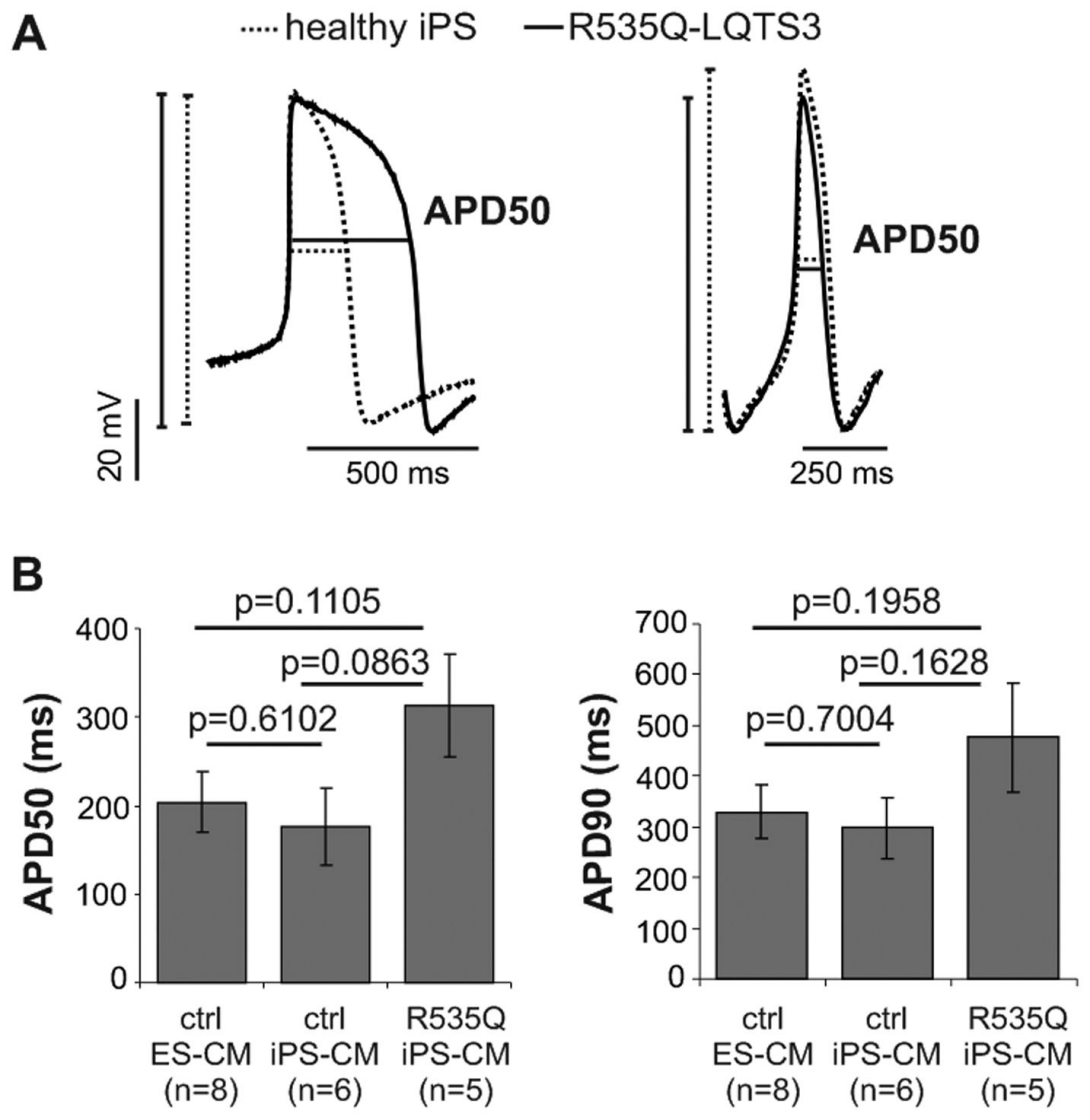
Patch clamp recordings of action potentials in R535Q-LQTS-3 iPS cell-derived CM. (A) Overlay of trace from R535Q-LQTS-3 and healthy cells representing the difference in action potential duration at 50% of repolarization (APD50) in ventricular (left) and atrial (right) like cells. (B) Statistical analysis of the differences in APD90 and APD50 of control and diseased cells.

LQTS-3 iPS cells from both patients differentiated into three cardiac subtypes as determined by their AP morphology and quantitative criteria (Table S1 in File S1). They were represented as ventricular-like subtype marked by plateau phase, atrial-like subtype marked by triangular shape and a pacemaker-like subtype marked by a comparatively slower diastolic depolarization velocity (Figure S7 in File S1). Because of insufficient data for ventricular-like CM from V240M-LQTS-3 iPS cells, statistical analysis of the AP parameters was possible only from R535Q-LQTS-3 iPS CM derived from patient NP0012. These analysis showed that the AP parameters, such as the overshoot, maximum diastolic potential (MDP), AP height, AP duration, AP frequency, upstroke rate (V_max_) and AP duration at 50% and 90% repolarization (APD50 and APD90), did not significantly differ between ventricular-like CM derived from R535Q-LQTS-3 iPS cells and control ES cell- and iPS cell-derived CM (Table 2). However, the mean APD50 values measured in R535Q-LQTS-3 CM were 54% and 78% longer than in control ES and iPS cell-derived CM, respectively (Figure 3A,B). The APD90 was also prolonged by 45% and 60% in R535Q-LQTS-3 CM when compared to control ES- and iPS-CM, respectively (Figure 3B). Although these differences did not reach statistical significance due to significant variations in AP duration among individual cells, the tendency for prolongation of APD50 and APD90 in LQTS-3 CM is in agreement with the expected electrophysiological phenotype of these cells. 

**Table 2:** Comparison of action potential parameters between control ES and iPS cell-derived ventricular-like cardiomyocytes and LQTS-3 iPS cell-derived ventricular-like cardiomyocytes carrying the mutation p.R535Q in sodium channel Na_v_1.5.

**Experimental group**	**BR (1/min)**	**Overshoot (mV)**	**MDP (mV)**	**Height (mV**)	**V_max_ (V/s)**	**APD90 (ms)**	**APD50 (ms)**
**Control ES-CM (n=8)**	88.29 ±18.09	44.26 ±4.04	-53.91 ±5.57	98.15 ±2.71	13.75 ±2.36	329.40 ±52.85	203.78 ±33.91
**Control iPS-CM (n=6)**	117.62 ±18.57	38.64 ±1.16	-63.58 ±2.30	102.22 ±1.94	24.11 ±4.84	297.70 ±60.53	175.45 ±43.25
**R535Q-LQTS-3 iPS-CM (n=5)**	104.25 ±30.17	46.70 ±3.11*	-54.86 ±3.03*	101.56 ±3.52	12.09 ±2.35	476.59 ±107.3	312.24 ±58.20

Abbreviations: BR – beating rate, MDP - maximum diastolic potential, V_max_ - maximum upstroke velocity, APD - action potential duration.

^*^ p<0.05 for comparison of LQTS3-iPS-CM and control iPS-CM using the two-tailed Student’s t-test. Statistical analysis of all other AP parameters did not show any significant difference between LQTS-3 iPS-CM and control ES-CM or iPS-CM.

### Sodium current properties in LQTS-3 CM

**Figure 4:**
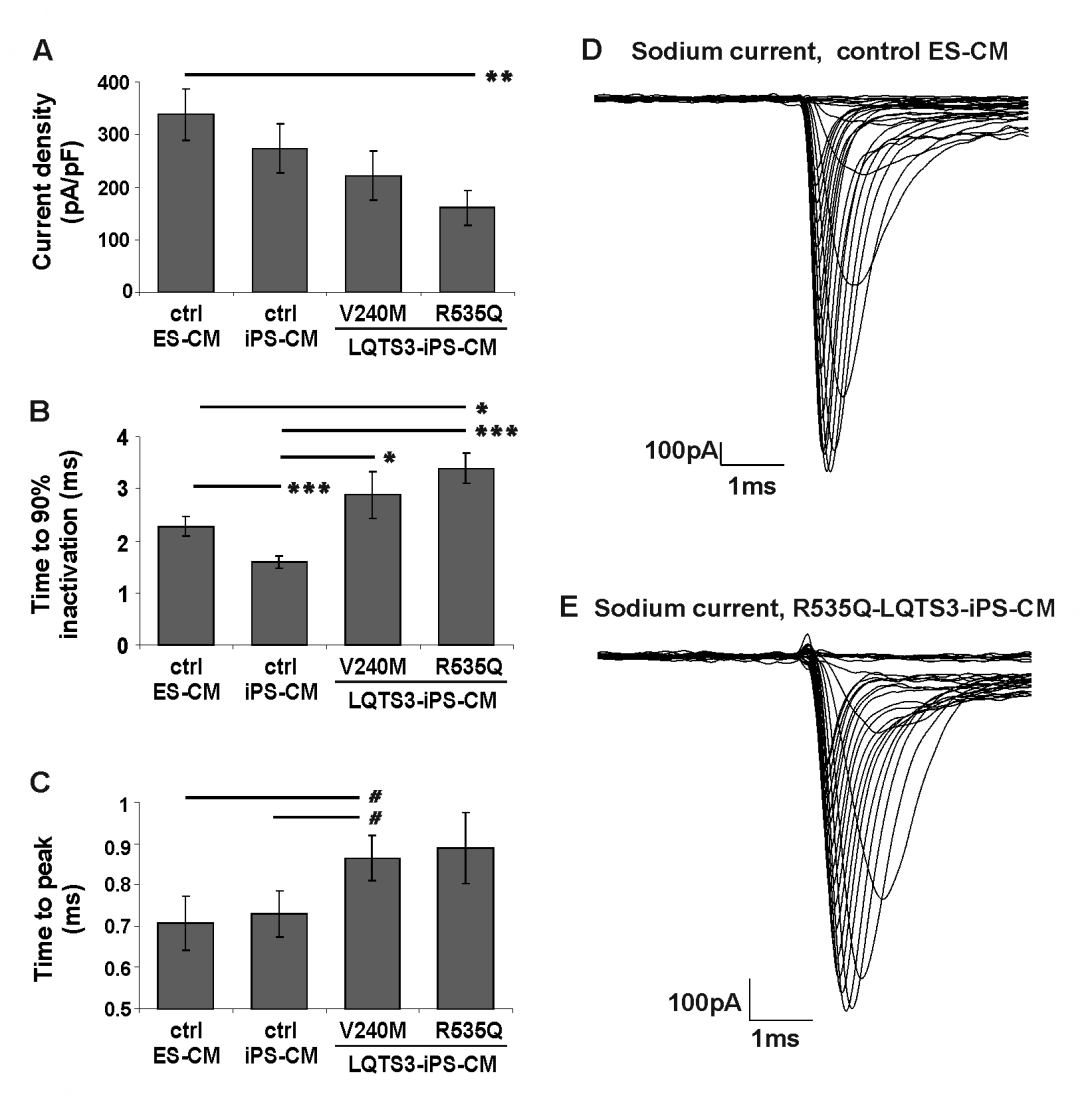
Sodium current measurements in control and LQTS-3 cells. Voltage-gated Na^+^ channel currents were recorded in a voltage-clamp mode. Values for current density (A), time of 90% inactivation (B) and time to peak (C) are shown for human ES cell derived CM (n=8), control iPS cell-derived CM (n=11) and for LQTS-3 iPS cell-derived CM from patients NP0012 (p.R535Q, n=13) and NP0016 (p.V240M, n=12). (D,E) Exemplary current traces with an increased persistent sodium current typical of LQTS-3 in diseased R535Q-LQTS-3 CM but not in healthy ES cell-derived CM. Symbols: # p<0.05, * p<0.02, ** p<0.01, ***p<0.005. Differences between all other groups were not statistically significant.

Next, we performed whole cell voltage-clamp studies on 30 day old CM derived from two LQTS-3 cell lines, one ES and one iPS control cell line to elaborate on the functional properties of Na^+^ channels. To estimate the expression of functional Na^+^ channels in the cell membrane, we calculated current densities in LQTS-3 CM and control ES-CM and iPS-CM by normalizing the maximal current amplitude to the cell size. Typical Na^+^ currents through voltage-gated Na^+^ channels could be recorded from all mutant and control cells (Figure 4). Na^+^ current density in iPS cell-derived CM from both patients showed a tendency to be smaller than that of CM derived from control cell lines at physiologically relevant depolarization steps (Figure 4A). The control ES and iPS cell-derived CM demonstrated a current density of 337±48 pA/pF (n=9) and 272±47 pA/pF (n= 11), respectively (Figure 4A). In contrast, the Na^+^ current density in LQTS-3 CM was 161±33 pA/pF for R535Q-LQTS-3 iPS cell-derived CM (n=13) and 221±47 pA/pF for V240M-LQTS-3 CM (n=12). However, the only difference reaching statistical significance was obtained only when R535Q-LQTS-3 CM were compared to ES cell-derived CM (p<0.01, Figure 4A), which may suggest that the effect of mutations on current density is weak. Moreover, the Na^+^ channels in CM from R535Q- and V240M-LQTS-3 iPS cells exhibited slower time to 90% inactivation (2.88±0.45 ms, n=12 and 3.39±0.29 ms, n=13, respectively) when compared to CM from control ES and iPS cell lines which inactivated significantly faster (2.27±0.18 ms, n= 8 and 1.59±0.12 ms, n=11, respectively; p<0.02 for R535Q-CM *vs* ES-CM; p<0.005 for R535Q-CM *vs* iPS-CM; p<0.02 for V240M *vs* iPS-CM; no significance for V240M-CM *vs* ES-CM comparison (Figure 4B). Similarly, time to peak was also significantly increased in V240M-LQTS-3 patient-derived CM as compared to control ES- and iPS-CM (p<0.05, Figure 4C). This parameter was also increased on R535Q-LQTS-3 CM, but this increase was not statistically significant due to a higher variability among cells. Taken together, these data suggest that the LQTS-3 CM have a defective Na^+^ channel which takes longer time for inactivation leading to persistent Na^+^ current (Figure 4D,E) and the tendency for prolongation of AP duration (Figure 3).

## Discussion

In this study, we investigated the utility of iPS cells for modelling the long QT syndrome type 3 caused by mutations located in the cytoplasmic loop between membrane-spanning regions S4 and S5 within DI (p.V240M) and in the intracellular linker region between DI and DII (p.R535Q) in the α-subunit of a voltage-gated sodium channel Na_v_1.5 encoded by a gene *SCN5A*. Both of these mutations have been previously described as possible LQTS-causing mutations in a genetic screen of 2500 unrelated cases referred for the LQTS genetic test [26]. Interestingly, the V240M mutation was also listed in association with BrS1 in the Inherited Arrhythmias Database (http://www.fsm.it/cardmoc/) as well as in the compendium of *SCN5A* mutations that were identified in 2111 patients referred for BrS genetic testing [27]. The V240M mutation was also found in three out of 78 patients with BrS1 in a recent study by Amin and coworkers [28]. However, this mutation has not been detected in 129 patients with possible BrS in the study of Crotti and coworkers [29] but was found in our LQTS patient NP0016 that did not meet the diagnostic criteria for BrS and in patients referred for the LQTS genetic testing in the study mentioned above [26]. Therefore, the mutation V240M can be considered as being most likely associated with the LQTS-3. 

Regarding the R535Q mutation, no evidence exists for its possible association with BrS1 or other arrhythmic syndromes. This mutation was not listed in the recently published compendium of BrS-associated *SCN5A* mutations identified in the above mentioned study [27] and it also does not appear on the list of BrS1-associated mutations in the Inherited Arrhythmias Database (http://www.fsm.it/cardmoc/). Instead, this database lists only the non-sense R535X mutation in *SCN5A* that is different from the missense R535Q mutation because it gives rise to a truncated protein in patients diagnosed with BrS [30,31]. Therefore, the available clinical evidence (including our patient NP0012) suggests that R535Q mutation is associated with LQTS-3.

A tightly regulated gating process is essential to ensure AP repolarization and termination in order to allow for propagation of the electrical signal. Sodium channel activation, open state inactivation and subsequent inactivation are the three important stages of channel activity. Mutations in the channel inactivation structures (DIII-DIV linker) have been reported to cause the vast majority of LQTS-3. The function of the DI-DII linker of the sodium channel, in which our mutations reside, in inactivation process is less understood, but mutations in this region (such as L619F) were shown to be associated with LQTS-3 [32]. 

All LQTS-3 mutations characterized so far lead to gain-of-function defects of the sodium channel resulting in a persistent current caused by continuous flow of Na^+^ through the inactivation-deficient channel during the plateau and repolarization phase of the AP [12,13,33,34]. Such a sustained current is expected to prolong the ventricular AP by directly counteracting the repolarizing K^+^ currents. The LQTS-3 patient-specific iPS cells generated in this study exhibited typical characteristics of pluripotent cells and could robustly differentiate into CM that recapitulated the disease phenotype in vitro. APD50 and APD90 in LQTS-3 CM averaged ^~^310 ms and ^~^480 ms, respectively, and were longer than those of control CMs (^~^200 ms and ^~^320 ms, respectively). However, in contrast to previous reports demonstrating significant prolongation of APD in iPS-CM derived from patients with LQT-1 and LQT-2 syndrome [14,15], the observed increase in APD50 and APD90 in our LQTS-3 iPS-CM was not statistically significant. The tendency toward prolongation of averaged APD90 without statistical significance was also reported by Malan et al. in murine LQTS-3 iPS-CM although the prolongation of APD90 in this study was detected when the AP duration was quantified in individual cardiomyocytes at slower pacing rates [13]. In contrast, the AP parameters were not measured in the study of Terrenoire and coworkers analyzing *de novo* SCN5A LQTS-3 mutation p.F1473C in human iPS-CM. These authors argued that the AP recordings would not be physiologically relevant in their cells due to their “relatively depolarized diastolic membrane potential that inactivates sodium channels and consequently minimize contributions of sodium channel activity to APs” [34]. Obviously, problems arising from immaturity of iPS-CM hinder the generation of statistically robust data in independent laboratories [35]. In addition, the lack of statistical significance also stems from technical difficulties in obtaining homogeneous data from a heterogeneous population of CM that typically emerge from iPS cells in vitro. In this study, some values differed by up to 10-fold among individual cells. This is in agreement with our previous study in which we have reported similar heterogeneity in Ca^2+^ current density between individual human iPS-CM [36] and with the study of Malan at al. in which high variability in APD90 between individual cells was also found [13]. This high cell-to-cell variability in electrophysiological parameters was most likely the result of intrinsic variability among CM in these cultures, which is a serious but frequently underestimated obstacle to the establishment of robust and faithful *in vitro* iPS cell-based disease models [35]. 

Our analyses of sodium channel properties were more conclusive and revealed that time-to-peak for sodium current and time to 90% of inactivation of the Na_v_1.5 were significantly longer in the LQTS-3 CM. This hints at defective biophysical properties of Na^+^ channel that is characteristic of LQTS-3. The reduced time-to-peak I_Na_ and inactivation rate measured under voltage clamp are expected to correlate with the upstroke rate (V_max_) measured under current clamp. In LQTS-3 CM the V_max_ was somewhat reduced (^~^12 V/s) compared to control ES-CM (^~^14 V/s) and iPS-CM (^~^24 V/s) but, also here, this differences were not statistically significant due to a high cell-to-cell and cell line-to-cell line variability. The sodium current density also tended to be smaller in both V240M- and R535Q-LQTS-3 CM compared to control cells but in overall this difference did not reach statistical significance. Therefore, we assume that on average the I_Na_ density is, if at all, only slightly reduced in LQTS-3 CM and that the gain-of-function defect leading to prolonged inactivation of Na_v_1.5, and tendency toward prolonged AP duration predominates in our cells. In addition, in some LQTS-3 iPS-CM we observed the late sodium current that we believe contributes to the observed tendency toward APD prolongation. Recent studies also showed that murine and human iPS-CM carrying different mutations in SCN5A have larger tetrodotoxin-sensitive persistent sodium current compared to wild type cells that was in the range of 0.5 to 1.7% of the peak I_Na_ [12,34]. Unfortunately, due to unstable measurements we were not able to statistically evaluate the classical persistent current in our cells. Additional analyses on larger data sets will be necessary to determine this parameter and elucidate the exact functional consequences of *SCN5A* mutations described in this study.

Polymorphisms in *SCN5A* amongst iPS cell lines are potential confounding factors that could differentially affect the expression and function of wild-type channels [23] and modulate properties of channels harboring LQTS-causing mutations [24,25]. One such common polymorphism is a variant allele c.1673AG leading to a substitution of amino acid histidine with arginine (p.H558R) in Na_v_1.5, which is present in about 21% of healthy individuals [23]. This polymorphism can restore the cell surface expression of a channel with an LQTS-3 causing mutation [37] and was shown to strongly reduce current density when coexpressed with the homozygous Q1077ins alternatively spliced variant of the sodium channel in a heterologous expression system [23]. However, the presence of H558R polymorphism in *SCN5A* is not considered a pathogenic mutation resulting in LQTS-3 and, hence, does not seem to have a major diagnostic or prognostic relevance in clinical practice [26]. Yet, it may still be possible that this or other non-pathogenic variants exert different modifying effects on channel function depending on coexisting disease-causing mutations in electrophysiological measurements with single cardiomyocytes. However, our control and LQTS-3 iPS cells carry the same heterozygous H558R polymorphism and, hence, this is not expected to interfere with our measurements. It has been also reported that there are two alternatively spliced sodium channel variants that are coexpressed in the heart cells of every individual and that they may exert differential effect on channel function in combination with H558R polymorphism [23]. One contains two glutamine residue at amino acid positions 1076 and 1077 (Q1077ins) and another, more predominant splice variant, contains only one glutamine at position 1076 (Q1077del). It is possible that relative ratios of these two variants in iPS cell-derived CM are different from those found in adult heart. However, since our control and patient-derived iPS cell lines and their derivatives most likely express the same mixture of short Q1077del and long Q1077ins alternatively spliced variants on the same background of the heterozygous H558R variant, we do not expect a significant confounding influence of these polymorphisms on Na_v_1.5 properties in our system. 

In summary, we showed that LQTS-3 patient-derived iPS-CM could recapitulate the disease phenotype *in vitro* but further improvements in generation and analysis of functionally homogeneous CM populations from iPS cells are necessary for establishment of robust *in vitro* iPS cell-based disease models for toxicity and efficacy testing in the drug discovery process. 

## Supporting Information

File S1 **The following files are included in File S1:** **Figure** **S1**, Schematic of voltage-gated sodium channel structure and location of mutations described in this study. **Figure** **S2**, ECG analysis of the two LQTS-3 patients. (A) ECG of patient NP0012 with heterozygous missense mutation c.1604G>A leading to point mutaiton p.R535Q. Corrected QT duration according to the Bazett's formula was about 540 ms. (B) ECG of patient NP0016 with heterozygous missense mutation c.718G>A leading to amino acid change p.V240M. Corrected QT duration according to the Bazett's formula was about 440 ms. ECG from leads V-V3 of both patient show presence of U wave following the T-wave. **Figure** **S3**, Detection of a heterozygous variant allele at the position c.1673A>G in *SCN5A* by direct sequencing of DNA isolated from control iPS cells and V240M-LQTS-3 and R535Q-LQTS-3 patients. This common polymorphism is found in 19-24% of individuals in a population and leads to a substitution of amino acid histidine (H) with arginine (R) (p.H558R) in Na_v_1.5. rs1805124 is a reference SNP number for this nonsynonymous single nucleotide polymorphism. **Figure** **S4**, Characterization of control and R535Q-LQTS-3 iPS cell lines. Flow cytometric analysis of SSEA4 and TRA-1-60 expression in R535Q-LQTS-3 iPS cell line derived from patient NP0012 (A) and in a control iPS cell line (B). Immunofluorescent images of OCT4, NANOG, TRA-1-80 and SSEA-4 expressing colonies of R535Q-LQTS-3 (C) and control (D) iPS cell lines. Nuclei were stained with DAPI (blue). **Figure** **S5**, Expression of transcription factors encoded by the reprogramming vector. (A-D) Quantitative RT-PCR analyses for exogenous *OCT3/4* (A), exogenous *SOX2* (B), exogenous *KLF4* (C) and exogenous *c-MYC* (D) in control iPS cells, R535Q-LQTS-3 iPS cells at passage 7 and human dermal fibroblasts from patient NP0012 at day 6 post transduction. Data are shown as mean of three technical replicates. **Figure** **S6**, Verification of SCN5A mutation in LQTS-3 iPS cells derived from patient NP0016. The presence of heterozygous missense mutation c.718G>A in *SCN5A* leading to amino acid change p.V240M in α subunit of Na_v_1.5 channel was confirmed by DNA sequencing. **Figure** **S7**, Representative traces of action potentials (APs) of atrial-like, ventricular-like and pacemaker/nodal-like cells obtained from control and V240M-LQTS-3 iPS cells. APs were analyzed in single cardiomyocytes by the whole-cell patch clamp method. The classification of different cardiac cell types into atrial-, ventricular- and pacemaker-like cells was based on the morphology of APs and AP parameters as summarized in the Table S1 in File S1. Black and red traces depict APs at different voltage scales as shown on the corresponding scale bars.
(PDF)
