# Supplementary material for: The Disease-Specific Phenotype in Cardiomyocytes Derived from Induced Pluripotent Stem Cells of Two Long QT Syndrome Type 3 Patients
Source: PLoS One. 2013 Dec 11;8(12):e83005. doi: 10.1371/journal.pone.0083005 (PMC3859612; doi:10.1371/journal.pone.0083005)
Supplement: File S1 — The following files are included in File S1: Figure S1, Schematic of voltage-gated sodium channel structure and location of mutations described in this study. Figure S2, ECG analysis of the two LQTS-3 patients. (A) ECG of patient NP0012 with heterozygous missense mutation c.1604G>A leading to point mutaiton p.R535Q. Corrected QT duration according to the Bazett's formula was about 540 ms. (B) ECG of patient NP0016 with heterozygous missense mutation c.718G>A leading to amino acid change p.V240M. Corrected QT duration according to the Bazett's formula was about 440 ms. ECG from leads V-V3 of both patient show presence of U wave following the T-wave. Figure S3, Detection of a heterozygous variant allele at the position c.1673A>G in SCN5A by direct sequencing of DNA isolated from control iPS cells and V240M-LQTS-3 and R535Q-LQTS-3 patients. This common polymorphism is found in 19-24% of individuals in a population and leads to a substitution of amino acid histidine (H) with arginine (R) (p.H558R) in Nav1.5. rs1805124 is a reference SNP number for this nonsynonymous single nucleotide polymorphism. Figure S4, Characterization of control and R535Q-LQTS-3 iPS cell lines. Flow cytometric analysis of SSEA4 and TRA-1-60 expression in R535Q-LQTS-3 iPS cell line derived from patient NP0012 (A) and in a control iPS cell line (B). Immunofluorescent images of OCT4, NANOG, TRA-1-80 and SSEA-4 expressing colonies of R535Q-LQTS-3 (C) and control (D) iPS cell lines. Nuclei were stained with DAPI (blue). Figure S5, Expression of transcription factors encoded by the reprogramming vector. (A-D) Quantitative RT-PCR analyses for exogenous OCT3/4 (A), exogenous SOX2 (B), exogenous KLF4 (C) and exogenous c-MYC (D) in control iPS cells, R535Q-LQTS-3 iPS cells at passage 7 and human dermal fibroblasts from patient NP0012 at day 6 post transduction. Data are shown as mean of three technical replicates. Figure S6, Verification of SCN5A mutation in LQTS-3 iPS cells derived from patient NP0016. Th [file pone.0083005.s001.pdf]

Supporting information

File S1 containing Table S1 and Figures S1-S7

The disease-specific phenotype in cardiomyocytes derived from induced pluripotent stem cells of two long QT syndrome type 3 patients

Fatima A., Kaifeng S., et al.

Table S1. Quantitative criteria used for classification of cardiomyocytes into ventricular-, atrial- and pacemaker/nodal-like subtypes.

	**Cardiomyocyte subtype**		
**AP-parameter**	**Ventricular-like**	**Atrial-like**	**Nodal-like**
**MDP**	<-60 mV	<-60 mV	>-60 mV
**APD90**	>150 ms	<150 ms	<150 ms
**APD90/APD50**	<1.6	>2.1	<2; >1.6
**V_{max}**	>20 V/s	>20 V/s	<10 V/s

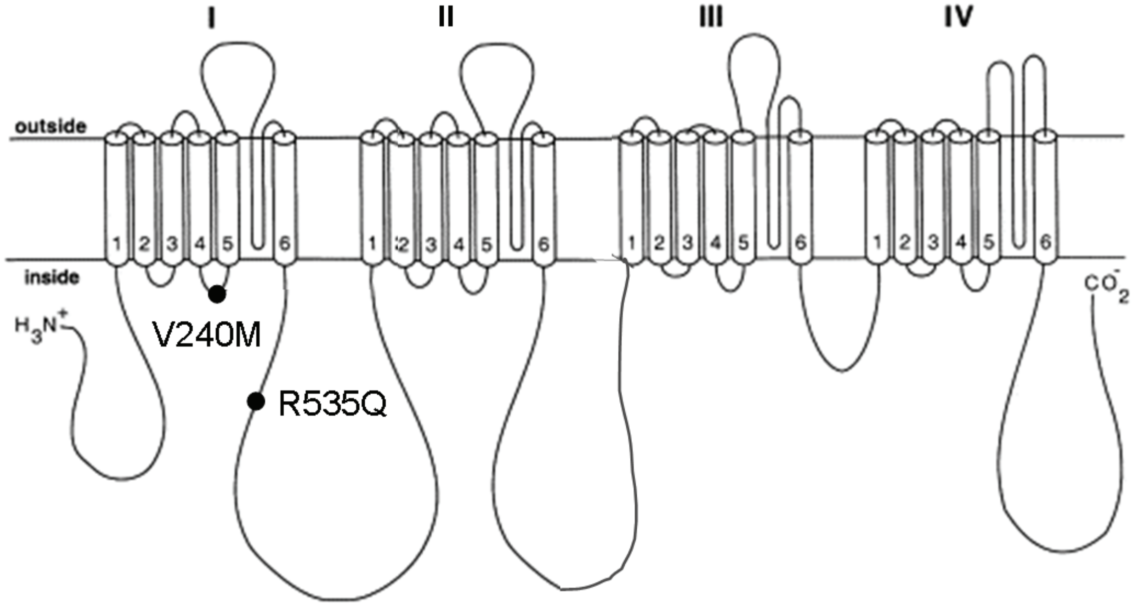

Figure S1. **Schematic of voltage-gated $Na_v1.5$ channel structure and location of mutations described in this study.**

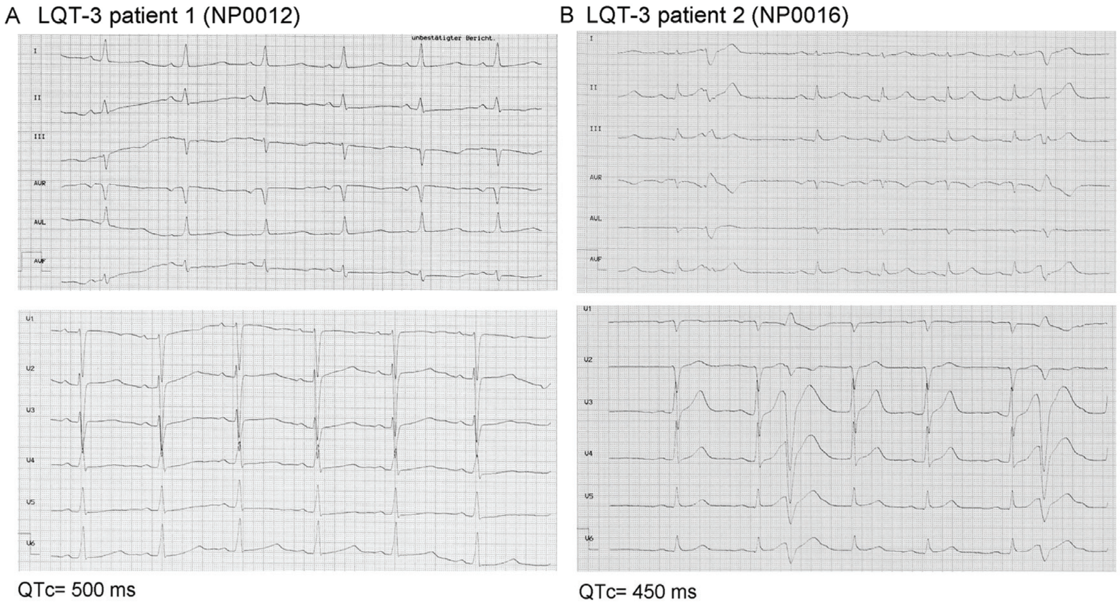

Figure S2. **ECG analysis of the two LQTS-3 patients.** (A) ECG of patient NP0012 with heterozygous missense mutation c.1604G>A leading to point mutation p.R535Q. Corrected QT duration according to the Bazett's formula was about 500 ms. (B) ECG of patient NP0016 with heterozygous missense mutation c.718G>A leading to amino acid change p.V240M. Corrected QT duration according to the Bazett's formula was about 450 ms. ECG from leads V-V3 of both patient show presence of U wave following the T-wave.

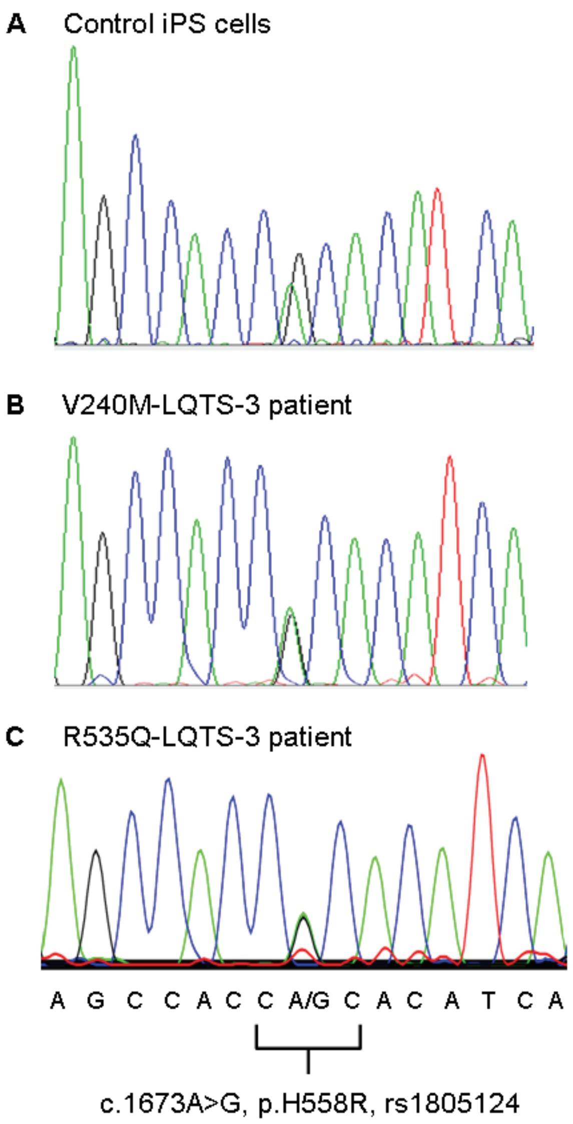

Figure S3. Detection of a heterozygous variant allele at the position c.1673A>G in *SCN5A* by direct sequencing of DNA isolated from control iPS cells and V240M-LQTS-3 and R535Q-LQTS-3 patients. This common polymorphism is found in 19-24% of individuals in a population and leads to a substitution of amino acid histidine (H) with arginine (R) (p.H558R) in $\mathrm{Na_v}$1.5, which may have a modifying effect on channel function. rs1805124 is a reference SNP number for this nonsynonymous single nucleotide polymorphism.

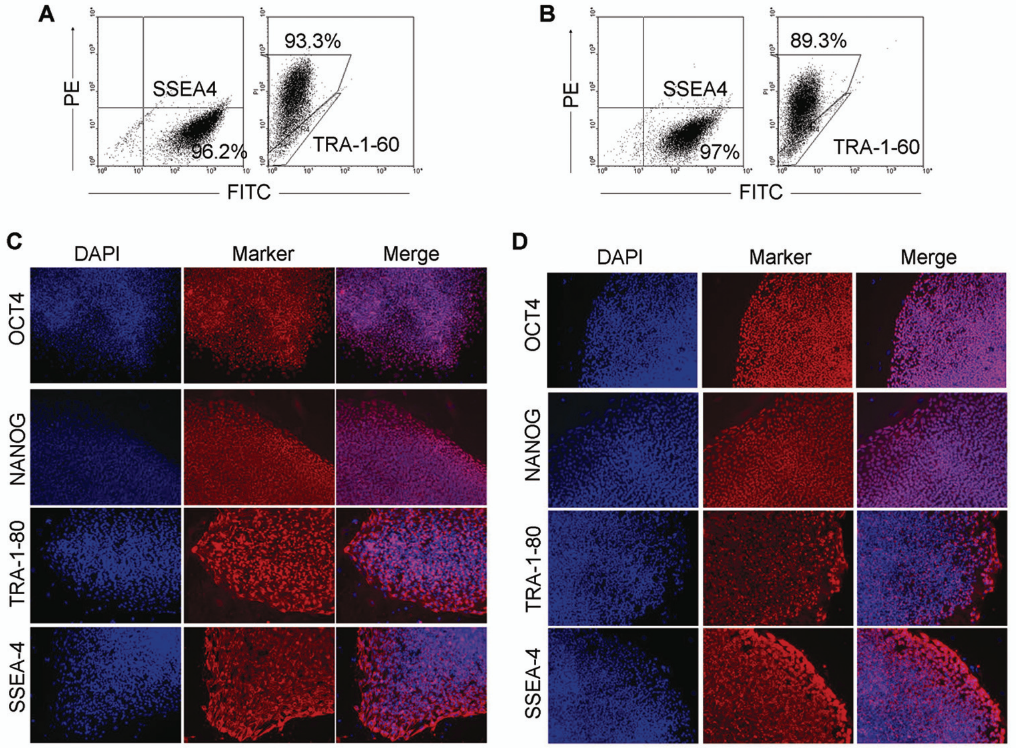

Figure S4. **Characterization of control and R535Q-LQTS-3 iPS cell lines.** Flow cytometric analysis of SSEA4 and TRA-1-60 expression in R535Q-LQTS-3 iPS cell line derived from patient NP0012 (A) and in control iPS cell line (B). Immunofluorescent images of OCT4, NANOG, TRA-1-80 and SSEA-4 expressing colonies of R535Q-LQTS-3 (C) and control (D) iPS cell lines. Nuclei were stained with DAPI (blue).

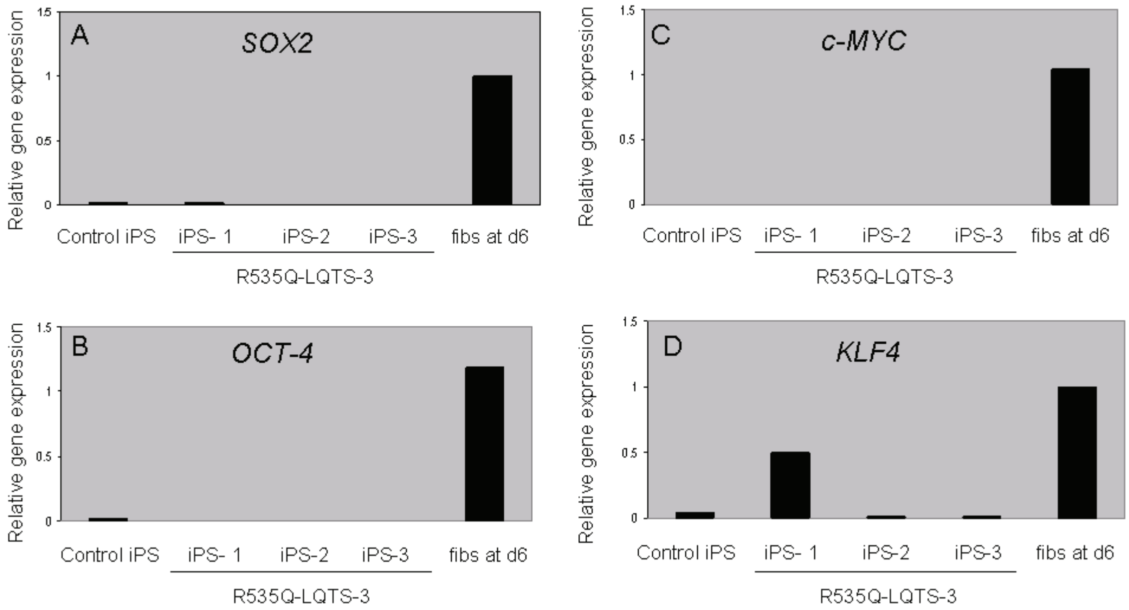

Figure S5. **Expression of transcription factors encoded by the reprogramming vector.** (A-D) Quantitative RT-PCR analyses for exogenous *SOX2* (A), exogenous *OCT3/4* (B), exogenous *c-MYC* (C) and exogenous *KLF4* (D) in control iPS cells, three different clones of R535Q-LQTS-3 iPS cell line at passage 7 and human dermal fibroblasts from patient NP0012 at day 6 post transduction. Data are shown as mean of three technical replicates.

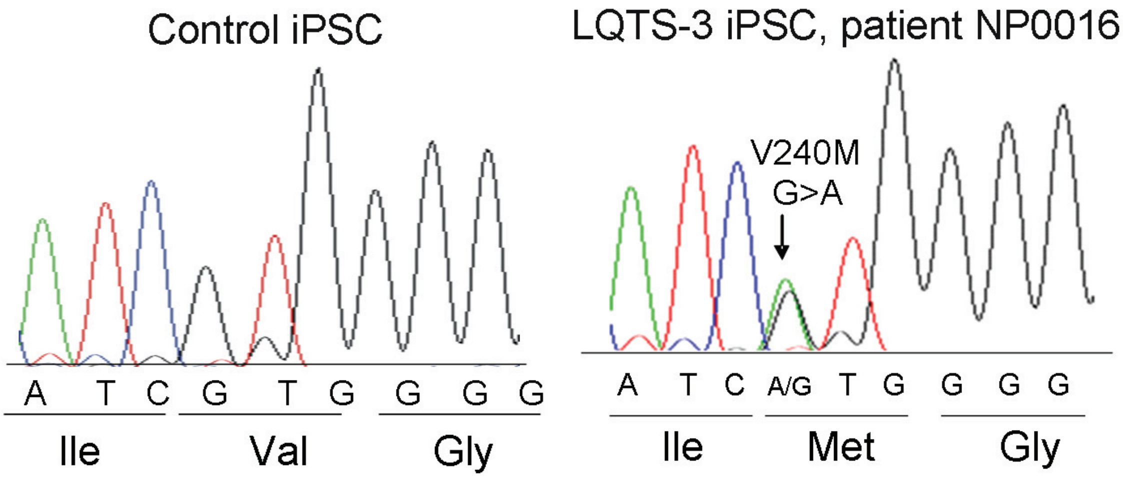

Figure S6. Verification of *SCN5A* mutation in LQTS-3 iPS cells derived from patient NP0016. The presence of heterozygous missense mutation c.718G>A in *SCN5A* leading to amino acid change p.V240M in α subunit of $Na_v1.5$ channel was confirmed by DNA sequencing.

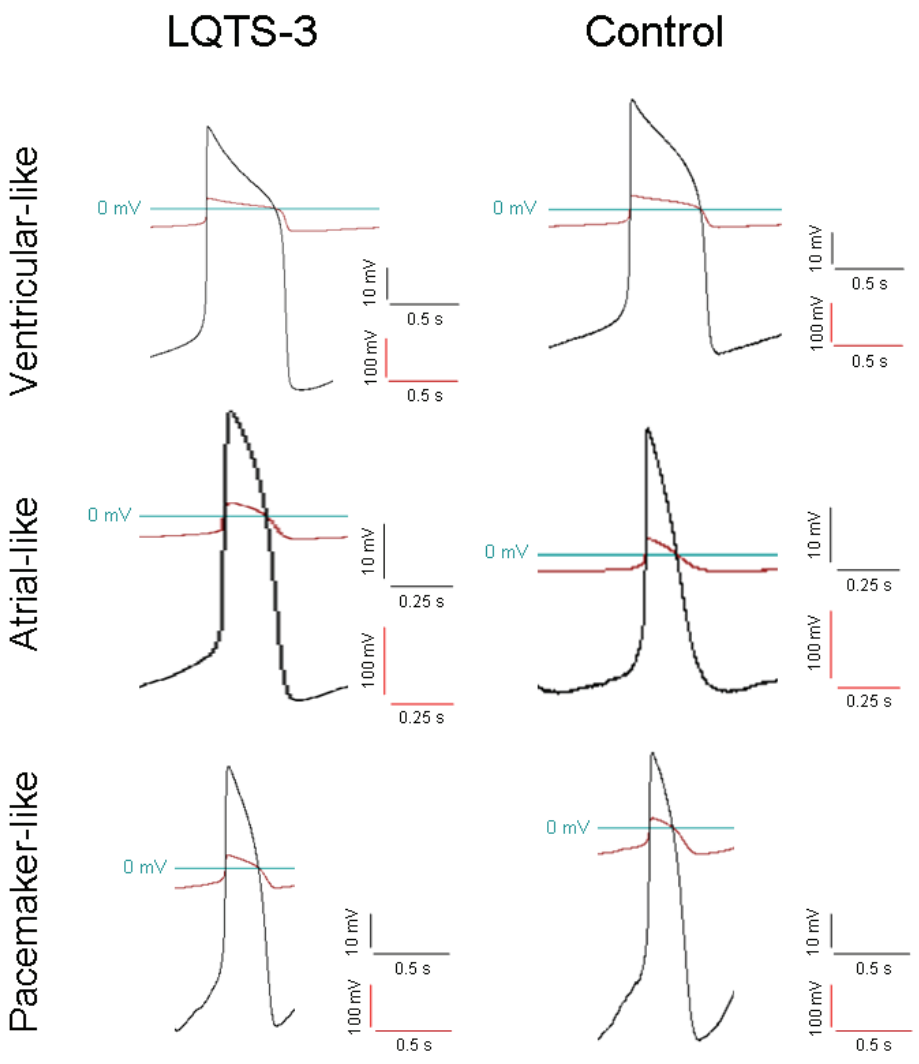

Figure S7. **Representative traces of action potentials (APs) of atrial-like, ventricular-like and pacemaker/nodal-like cells obtained from control and V240M-LQTS-3 iPS cells**. APs were analyzed in single cardiomyocytes by the whole-cell patch clamp method. The classification of different cardiac cell types into atrial-, ventricular- and pacemaker-like cells was based on the morphology of APs and AP parameters as summarized in the Table S1. Black and red traces depict APs at different voltage scales as shown on the corresponding scale bars.
